# OBJECTIVE AND SUBJECTIVE INDICATORS OF CAREGIVER BURDEN FOLLOWING AN AMYLOID-Β PET SCAN: A MIXED-METHODS STUDY

**DOI:** 10.1093/geroni/igae098.1624

**Published:** 2024-12-31

**Authors:** Elyse Couch

**Affiliations:** Brown University, Providence, Rhode Island, United States

## Abstract

Dementia caregiving is often associated with high levels of burden which can lead to negative outcomes. Caregiver burden consists of disease-specific and perceived stressors - or objective and subjective indicators of burden. Amyloid-β positron emission tomography (PET) scans can be used to enhance the accuracy of dementia diagnoses. However, the relationship between amyloid scan results and caregiver burden is less well understood. We conducted a parallel mixed-methods study. We used quantitative survey data from 1,338 care partners to persons with MCI and dementia who received an amyloid scan as part of the CARE-IDEAS study in the US. Logistic regression models were used to test the association between the scan result and objective measures of caregiver burden. Semi-structured interviews were conducted with a subsample of 62 care partners. Thematic analysis was used to investigate subjective indicators by exploring care partners’ perceptions of their role following an amyloid scan. Quantitative analysis showed that an elevated amyloid scan result was associated with spending more hours caregiving for MCI care partners. However, our thematic analysis showed that the scan result influenced participants’ perceptions and expectations of their caregiving role and helped some care partners to develop coping strategies. All care partners described needing practical and emotional support. Amyloid scan results can influence objective and subjective indicators of burden and may present an opportunity for diagnosing clinicians to identify and address care partners’ emotional and practical support needs.

